# Contributions of binocular and monocular cues to motion-in-depth perception

**DOI:** 10.1167/19.3.2

**Published:** 2019-03-05

**Authors:** Lowell Thompson, Mohan Ji, Bas Rokers, Ari Rosenberg

**Affiliations:** ari.rosenberg@wisc.edu; Department of Psychology, University of Wisconsin–Madison, Madison, WI, USA; Department of Neuroscience, School of Medicine and Public Health, University of Wisconsin–Madison, Madison, WI, USA; Department of Psychology, University of Wisconsin–Madison, Madison, WI, USA; Department of Psychology, University of Wisconsin–Madison, Madison, WI, USA; Department of Neuroscience, School of Medicine and Public Health, University of Wisconsin–Madison, Madison, WI, USA

**Keywords:** *motion-in-depth*, *psychophysics*, *binocular*, *monocular*, *3D*

## Abstract

Intercepting and avoiding moving objects requires accurate motion-in-depth (MID) perception. Such motion can be estimated based on both binocular and monocular cues. Because previous studies largely characterized sensitivity to these cues individually, their relative contributions to MID perception remain unclear. Here we measured sensitivity to binocular, monocular, and combined cue MID stimuli using a motion coherence paradigm. We first confirmed prior reports of substantial variability in binocular MID cue sensitivity across the visual field. The stimuli were matched for eccentricity and speed, suggesting that this variability has a neural basis. Second, we determined that monocular MID cue sensitivity also varied considerably across the visual field. A major component of this variability was geometric: An MID stimulus produces the largest motion signals in the eye contralateral to its visual field location. This resulted in better monocular discrimination performance when the contralateral rather than ipsilateral eye was stimulated. Third, we found that monocular cue sensitivity generally exceeded, and was independent of, binocular cue sensitivity. Finally, contralateral monocular cue sensitivity was found to be a strong predictor of combined cue sensitivity. These results reveal distinct factors constraining the contributions of binocular and monocular cues to three-dimensional motion perception.

## Introduction

Accurate motion-in-depth (MID) perception is required to intercept and avoid objects. The direction of MID (i.e., “toward” vs. “away”) is conveyed by signals contained within time-varying retinal images. These signals can be broadly divided into binocular cues which require comparisons of information across the two eyes, and monocular cues which include information available to a single eye.

Binocular cues to MID include interocular velocity differences (IOVD) and changing disparity (CD; Allen, Haun, Hanley, Green, & Rokers, 2015; Beverley & Regan, 1973; Brooks, 2002; Cumming & Parker, 1994; Czuba, Rokers, Huk, & Cormack, 2012; Joo, Czuba, Cormack, & Huk, 2016; Lages & Heron, 2010; Nefs, O'Hare, & Harris, 2010; Norcia & Gerhard, 2015). Previous work revealed considerable variability in binocular MID cue sensitivity across the visual field of individual observers (Barendregt, Dumoulin, & Rokers, 2014; Hong & Regan, 1989; Richards & Regan, 1973).

Monocular cues to MID are provided by optic flow, as well as changes in the retinal size and density of visual elements (Longuet-Higgins & Prazdny, 1980; Regan & Beverley, 1979). Whereas binocular MID cues are often studied using stimuli that simulate motion through relatively confined regions of three-dimensional (3D) space, monocular MID cues have mostly been studied in the context of self-motion, using stimuli that cover large portions of the visual field (Duffy & Wurtz, 1991a; Rutschmann, Schrauf, & Greenlee, 2000; Warren, 2004). As such, variability in monocular MID cue sensitivity across the visual field has not been systematically characterized. Furthermore, many studies used monocular cue stimuli that assumed a “cyclopean eye,” and simultaneously presented the same optic flow pattern to both eyes (Cottereau et al., 2017; de Jong, Shipp, Skidmore, Frackowiak, & Zeki, 1994; Duffy & Wurtz, 1991a; Graziano, Andersen, & Snowden, 1994; Mineault, Khawaja, Butts, & Pack, 2012; Morrone et al., 2000; Uesaki & Ashida, 2015; Xu, Wallisch, & Bradley, 2014) The monocular information received by each eye differs under natural viewing conditions (Cormack, Czuba, Knoll, & Huk, 2017), but the extent to which the differences affect MID perception has not been investigated.

Here we measured MID sensitivity by asking observers to perform a “toward”/“away” discrimination task in which motion coherence was varied. The stimuli simulated dots moving through small volumes of 3D space, and selectively isolated either binocular or monocular MID cues, or contained both cues. We found considerable variability in performance across the visual field, which reflected different factors for binocular and monocular cues. Variability in sensitivity to binocular cues existed across eccentricity- and speed-matched stimuli, suggesting a neural basis. Sensitivity to monocular cues depended on whether the stimulus was in the contralateral or ipsilateral visual field relative to the stimulated eye. Variability in monocular MID cue sensitivity thus reflected geometric consequences of the horizontal offset between the eyes. This result highlights that stimuli in the 3D world create distinct signals for each eye. Consequently, estimates of cue sensitivity measured by presenting identical stimuli to both eyes simultaneously may not accurately reflect monocular cue sensitivity under natural conditions. We further found that monocular cue sensitivity generally exceeded, and was statistically independent of, binocular cue sensitivity. Lastly, we found that contralateral monocular cue sensitivity was a strong predictor of combined cue sensitivity. In sum, the present results identify distinct factors constraining 3D motion perception, and have fundamental implications for our understanding of how signals from the two eyes are combined to achieve motion perception in the 3D world.

## Methods

### Observers

Seven observers (three male; four female) participated after providing informed consent. All observers had normal or corrected-to-normal vision. Two observers were authors, and two others had extensive psychophysical experience. Experimental procedures were approved by the University of Wisconsin–Madison Institutional Review Board and carried out in accordance with the Declaration of Helsinki.

### Apparatus and display

Stimuli were presented on a 23-in. Planar SA2311W LED monitor (120 Hz refresh rate, 1920 × 1080 pixels resolution) at a viewing distance of 90 cm. Images were temporally interleaved and presented separately to each eye at 60 Hz using an NVIDIA 3D Vision 2 Wireless Glasses Kit (NVIDIA Corporation). This kit uses active polarizers (i.e., shutters), and had an average cross talk of 1.5% (when minimum luminance was presented to the “open” eye, and maximum luminance was presented to the “closed” eye). Observers used a chin rest to maintain a stable head position. Stimuli were rendered in MATLAB (R2015a; MathWorks, Natick, MA) using the Psychophysics Toolbox 3 (Brainard, 1997; Kleiner et al., 2007; Pelli, 1997).

### Stimuli

**Figure 1:**
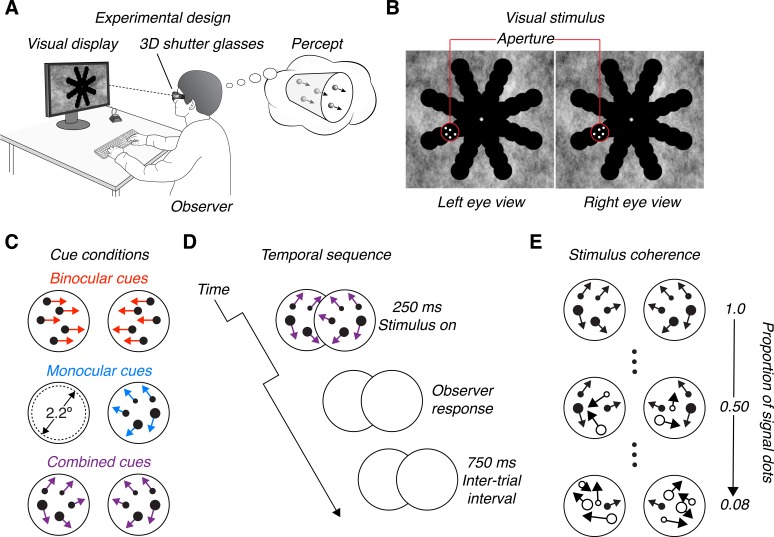
Schematic of the display, stimulus, and conditions. (A) Experimental design: Observers viewed a visual display through 3D shutter glasses. Stimuli depicted dots moving in depth through a cylindrical volume oriented perpendicular to the display. Observers reported the perceived motion direction (toward/away). (B) Visual stimulus: On each trial, a stimulus appeared in one aperture (e.g., the one outlined in red for illustration). Observers fixated a central target (a white dot). A 1/f noise background pattern facilitated stable version and vergence. (C) Cue conditions: On each trial, one of three cue conditions was presented. Binocular cue stimuli contained opposite horizontal motions in the two eyes. Monocular cue stimuli were optic flow patterns shown to one eye. Combined cue stimuli were optic flow patterns shown to both eyes, and thus contained both cues. (D) Temporal sequence: Stimuli were presented for 250 ms. After responding, there was an inter-trial interval of 750 ms. (E) Stimulus coherence: Stimuli were presented at five motion coherences (proportion of signal dots: 0.08, 0.25, 0.50, 0.75, and 1). Signal dots (depicted here as filled black dots) moved either toward or away from the observer. Noise dots (depicted here as open circles) moved to random locations, producing motion signals of random speed and direction. The number of dots, their sizes, and the depicted optic flow patterns used in the schematics were selected to convey the MID cues, rather than portray the actual stimuli. Example stimuli are shown in the Supplementary Movies.

We presented random dot MID stimuli that moved perpendicular to the plane of the monitor, either toward or away from the observer. Stimuli were presented at eight equally spaced visual field locations (polar angles between 22.5° and 337.5° in 45° steps), with an eccentricity of 4.5° from fixation (Figure 1A and B). They were presented within 2.5° diameter apertures. On each trial, an MID stimulus was presented at one pseudorandomly chosen location.

A stimulus consisted of 12 light (9.90 cd/m^2^) dots on a dark (0.04 cd/m^2^) background. Each dot subtended 0.1° of visual angle at screen distance. Dots were initialized with random *x*, *y*, and *z* positions and moved within a cylindrical volume perpendicular to the screen. This cylinder extended ±0.3° of horizontal disparity, which corresponded to a depth range of ∼14 cm for the average observer. The stimulus duration was 250 ms. Each dot traversed half the volume (0.3° of disparity) in this time, corresponding to a world speed of ∼28 cm/s. If a dot reached a disparity of ±0.3°, it wrapped to the opposite end of the volume. Dot wrapping could cause an apparent MID signal in the direction opposite of the intended stimulus direction. To reduce this effect, a dot was assigned new *x* and *y* positions when it wrapped. To ensure that binocular correspondence was not interrupted due to occlusion of a dot by the edge of the aperture, the *x* and *y* coordinates were restricted to lie within the central 2.2° of the aperture (Figure 1C). Observers were given unlimited time to respond. A new trial began 750 ms after a response.

To assess MID sensitivity, we manipulated motion coherence by varying the proportion of signal to noise dots. Since we used shutter glasses, the images presented to the two eyes were temporally interleaved. Stimuli were rendered as a series of stereoscopic (right-left) frame-pairs in which both the right and left eye images were presented before the dot positions were updated. For each stereoscopic frame-pair, we randomly selected a subset of dots as signal dots which moved either toward or away from the observer (perpendicular to the screen). The remaining dots (noise dots) were given random *x, y,* and *z* coordinates. This procedure ensured that coherence levels were commensurate across cue conditions, and reduced the likelihood of observers tracking individual dots. Five coherence levels (proportion of signal dots) were tested: 0.08, 0.25, 0.50, 0.75, and 1 (1/12, 3/12, 6/12, 9/12, and 12/12 signal/total dots, respectively). Visual field location, cue condition, MID direction, and coherence were chosen pseudorandomly each trial.

To facilitate version and vergence, we presented a 1/*f* noise background. As in earlier work (Barendregt, Dumoulin, & Rokers, 2014, 2016), the background included 40 apertures arranged in a “spoke-wheel” pattern which further aided binocular fusion (Figure 1A and B). The diameter of each aperture was 2.5°. The polar angles were spaced between 22.5° and 337.5° in 45° steps. The eccentricities were linearly spaced between 1.5° and 7.5° in 1.5° steps. MID stimuli were only presented in apertures at 4.5° eccentricity. The texture of the noise pattern changed each session. A fixation point was presented in the middle of the screen. We instructed observers to maintain fixation on this point throughout the duration of each trial, but did not enforce fixation. We encouraged fixation by having the stimuli appear briefly (250 ms) in pseudorandom locations. The noise background, apertures, and fixation point were visible at all times.

We assessed sensitivity to stimuli for which the direction of MID was signaled by monocular and binocular cues (combined cue stimuli), monocular cues only, and binocular cues only. For combined cue stimuli, a combination of projective geometry and stereoscopic presentation was used to achieve congruent monocular and binocular cues (see Supplementary Movie 1). To the observers, the stimuli appeared as a cloud of dots with some (signal) dots moving perpendicular to the screen and other (noise) dots moving randomly. Similar configurations are routinely used to study heading perception, with the exception that our stimuli were presented within small apertures rather than over a large region of the visual field. As a consequence of projective geometry, changes in retinal dot size and the pattern of optic flow provided monocular MID cues. At the nearest and furthest depth planes, the dot sizes were ∼1.7 mm and ∼1.5 mm on the screen, respectively. Given that individual dots only traversed half the volume (making the average size change ∼0.12 mm), we believe it is unlikely that changes in dot size were the major source of monocular MID information. Instead, this information was likely provided by the pattern of optic flow. As a consequence of stereoscopic rendering, IOVD and CD signals provided binocular MID cues (Allen et al., 2015; Beverley & Regan, 1973; Brooks, 2002; Cumming & Parker, 1994; Czuba et al., 2012; Joo et al., 2016; Lages & Heron, 2010; Nefs et al., 2010; Norcia & Gerhard, 2015). Prior work using planar MID stimuli found that observers tend to rely on IOVD cues more than CD cues to judge MID (Allen et al., 2015). Thus, IOVD cues likely provided the major source of binocular MID information.

For the monocular cue only stimuli, binocular cues were eliminated by presenting single eye views of the combined cue stimuli (see Supplementary Movies 2, 3). The noise background and fixation point were visible to both eyes. The pattern of optic flow depended on the direction of MID, the visual field location, and the stimulated eye (pseudorandomly selected each trial). Motion towards (or away from) an observer produces a pattern of retinal motion that expands or contracts (Longuet-Higgins & Prazdny, 1980). Thus, the direction of retinal motion at a given visual field location depends on the polar angle between the focus of expansion (contraction) and that location. In addition, due to the horizontal offset between the eyes, MID produces different right and left eye optic flow patterns (Cormack et al., 2017).

For the binocular cue only stimuli, monocular cues that signal MID were eliminated by (a) using orthographic projection to remove perspective cues, (b) horizontally translating the right and left eye dot pairs with equal and opposite speeds (0.6°/s) regardless of the visual field location, and (c) drawing the dots with a fixed size (0.1° of visual angle) regardless of the simulated distance (see Supplementary Movie 4). The binocular cue stimuli thus contained IOVD and CD cues to MID. Since the retinal dot sizes and densities were constant, the stimuli contained no perspective information capable of signaling the direction of MID. This rendering approach is comparable to those used in previous MID studies (Allen et al., 2015; Barendregt et al., 2014, 2016; Czuba, Rokers, Huk, & Cormack, 2010), with the exception that we simulated a volume rather than a plane of dots.

### Procedure

Each session, observers viewed all combinations of the eight visual field locations, three cue conditions, two MID directions, and five motion coherences (8 × 3 × 2 × 5 = 240 unique stimuli) six times (1,440 trials/session). To prevent observers from relying on one cue over the other due to exposure differences, we equated the number of binocular, monocular, and combined cue presentations. For monocular cue stimuli, this meant that each eye saw half the total number of presentations (i.e., the monocular cue stimuli were presented three times to each eye every session). All observers completed six sessions (*N* = 8,640 trials). Observers reported the direction of motion for each trial using the up (“away”) and down (“toward”) arrow keys on a computer keyboard. No feedback was provided.

### Data analysis

For each visual field location and cue condition, we calculated the proportion of “toward” responses *g*(*x*) as a function of direction and motion coherence *x*. We then fit *g*(*x*) with a cumulative Gaussian, allowing for a nonzero lapse rate to account for the possibility of nonperceptual errors (e.g., “click error” or missing the stimulus due to blinking; Klein, 2001), using maximum likelihood estimation in MATLAB:

\newcommand{\bialpha}{\boldsymbol{\alpha}}\newcommand{\bibeta}{\boldsymbol{\beta}}\newcommand{\bigamma}{\boldsymbol{\gamma}}\newcommand{\bidelta}{\boldsymbol{\delta}}\newcommand{\bivarepsilon}{\boldsymbol{\varepsilon}}\newcommand{\bizeta}{\boldsymbol{\zeta}}\newcommand{\bieta}{\boldsymbol{\eta}}\newcommand{\bitheta}{\boldsymbol{\theta}}\newcommand{\biiota}{\boldsymbol{\iota}}\newcommand{\bikappa}{\boldsymbol{\kappa}}\newcommand{\bilambda}{\boldsymbol{\lambda}}\newcommand{\bimu}{\boldsymbol{\mu}}\newcommand{\binu}{\boldsymbol{\nu}}\newcommand{\bixi}{\boldsymbol{\xi}}\newcommand{\biomicron}{\boldsymbol{\micron}}\newcommand{\bipi}{\boldsymbol{\pi}}\newcommand{\birho}{\boldsymbol{\rho}}\newcommand{\bisigma}{\boldsymbol{\sigma}}\newcommand{\bitau}{\boldsymbol{\tau}}\newcommand{\biupsilon}{\boldsymbol{\upsilon}}\newcommand{\biphi}{\boldsymbol{\phi}}\newcommand{\bichi}{\boldsymbol{\chi}}\newcommand{\bipsi}{\boldsymbol{\psi}}\newcommand{\biomega}{\boldsymbol{\omega}}\begin{equation}\tag{1}g\left( x \right) = {\lambda } + \left( {1 - 2\lambda } \right){{1} \over 2}\left[ {1 + {\it{erf}}\left( {{{x - {\mu }} \over {{\sigma }\sqrt 2 }}} \right)} \right],\!\end{equation}

where $\newcommand{\bialpha}{\boldsymbol{\alpha}}\newcommand{\bibeta}{\boldsymbol{\beta}}\newcommand{\bigamma}{\boldsymbol{\gamma}}\newcommand{\bidelta}{\boldsymbol{\delta}}\newcommand{\bivarepsilon}{\boldsymbol{\varepsilon}}\newcommand{\bizeta}{\boldsymbol{\zeta}}\newcommand{\bieta}{\boldsymbol{\eta}}\newcommand{\bitheta}{\boldsymbol{\theta}}\newcommand{\biiota}{\boldsymbol{\iota}}\newcommand{\bikappa}{\boldsymbol{\kappa}}\newcommand{\bilambda}{\boldsymbol{\lambda}}\newcommand{\bimu}{\boldsymbol{\mu}}\newcommand{\binu}{\boldsymbol{\nu}}\newcommand{\bixi}{\boldsymbol{\xi}}\newcommand{\biomicron}{\boldsymbol{\micron}}\newcommand{\bipi}{\boldsymbol{\pi}}\newcommand{\birho}{\boldsymbol{\rho}}\newcommand{\bisigma}{\boldsymbol{\sigma}}\newcommand{\bitau}{\boldsymbol{\tau}}\newcommand{\biupsilon}{\boldsymbol{\upsilon}}\newcommand{\biphi}{\boldsymbol{\phi}}\newcommand{\bichi}{\boldsymbol{\chi}}\newcommand{\bipsi}{\boldsymbol{\psi}}\newcommand{\biomega}{\boldsymbol{\omega}}\mu$ is the estimate of observer bias, $\newcommand{\bialpha}{\boldsymbol{\alpha}}\newcommand{\bibeta}{\boldsymbol{\beta}}\newcommand{\bigamma}{\boldsymbol{\gamma}}\newcommand{\bidelta}{\boldsymbol{\delta}}\newcommand{\bivarepsilon}{\boldsymbol{\varepsilon}}\newcommand{\bizeta}{\boldsymbol{\zeta}}\newcommand{\bieta}{\boldsymbol{\eta}}\newcommand{\bitheta}{\boldsymbol{\theta}}\newcommand{\biiota}{\boldsymbol{\iota}}\newcommand{\bikappa}{\boldsymbol{\kappa}}\newcommand{\bilambda}{\boldsymbol{\lambda}}\newcommand{\bimu}{\boldsymbol{\mu}}\newcommand{\binu}{\boldsymbol{\nu}}\newcommand{\bixi}{\boldsymbol{\xi}}\newcommand{\biomicron}{\boldsymbol{\micron}}\newcommand{\bipi}{\boldsymbol{\pi}}\newcommand{\birho}{\boldsymbol{\rho}}\newcommand{\bisigma}{\boldsymbol{\sigma}}\newcommand{\bitau}{\boldsymbol{\tau}}\newcommand{\biupsilon}{\boldsymbol{\upsilon}}\newcommand{\biphi}{\boldsymbol{\phi}}\newcommand{\bichi}{\boldsymbol{\chi}}\newcommand{\bipsi}{\boldsymbol{\psi}}\newcommand{\biomega}{\boldsymbol{\omega}}\sigma$ reflects the precision of the responses, and *λ* is the lapse rate. To stabilize fits when precision was low, we enforced a motion coherence bound of ±0.50 on $\newcommand{\bialpha}{\boldsymbol{\alpha}}\newcommand{\bibeta}{\boldsymbol{\beta}}\newcommand{\bigamma}{\boldsymbol{\gamma}}\newcommand{\bidelta}{\boldsymbol{\delta}}\newcommand{\bivarepsilon}{\boldsymbol{\varepsilon}}\newcommand{\bizeta}{\boldsymbol{\zeta}}\newcommand{\bieta}{\boldsymbol{\eta}}\newcommand{\bitheta}{\boldsymbol{\theta}}\newcommand{\biiota}{\boldsymbol{\iota}}\newcommand{\bikappa}{\boldsymbol{\kappa}}\newcommand{\bilambda}{\boldsymbol{\lambda}}\newcommand{\bimu}{\boldsymbol{\mu}}\newcommand{\binu}{\boldsymbol{\nu}}\newcommand{\bixi}{\boldsymbol{\xi}}\newcommand{\biomicron}{\boldsymbol{\micron}}\newcommand{\bipi}{\boldsymbol{\pi}}\newcommand{\birho}{\boldsymbol{\rho}}\newcommand{\bisigma}{\boldsymbol{\sigma}}\newcommand{\bitau}{\boldsymbol{\tau}}\newcommand{\biupsilon}{\boldsymbol{\upsilon}}\newcommand{\biphi}{\boldsymbol{\phi}}\newcommand{\bichi}{\boldsymbol{\chi}}\newcommand{\bipsi}{\boldsymbol{\psi}}\newcommand{\biomega}{\boldsymbol{\omega}}\mu$. We assumed a maximum lapse rate of 2%. Sensitivity ($\newcommand{\bialpha}{\boldsymbol{\alpha}}\newcommand{\bibeta}{\boldsymbol{\beta}}\newcommand{\bigamma}{\boldsymbol{\gamma}}\newcommand{\bidelta}{\boldsymbol{\delta}}\newcommand{\bivarepsilon}{\boldsymbol{\varepsilon}}\newcommand{\bizeta}{\boldsymbol{\zeta}}\newcommand{\bieta}{\boldsymbol{\eta}}\newcommand{\bitheta}{\boldsymbol{\theta}}\newcommand{\biiota}{\boldsymbol{\iota}}\newcommand{\bikappa}{\boldsymbol{\kappa}}\newcommand{\bilambda}{\boldsymbol{\lambda}}\newcommand{\bimu}{\boldsymbol{\mu}}\newcommand{\binu}{\boldsymbol{\nu}}\newcommand{\bixi}{\boldsymbol{\xi}}\newcommand{\biomicron}{\boldsymbol{\micron}}\newcommand{\bipi}{\boldsymbol{\pi}}\newcommand{\birho}{\boldsymbol{\rho}}\newcommand{\bisigma}{\boldsymbol{\sigma}}\newcommand{\bitau}{\boldsymbol{\tau}}\newcommand{\biupsilon}{\boldsymbol{\upsilon}}\newcommand{\biphi}{\boldsymbol{\phi}}\newcommand{\bichi}{\boldsymbol{\chi}}\newcommand{\bipsi}{\boldsymbol{\psi}}\newcommand{\biomega}{\boldsymbol{\omega}}1/\sigma$) was our primary measure of interest.

To determine the sensitivity level expected from chance, we simulated the performance of an observer who completed the six sessions by always responding randomly. We then bootstrapped the sensitivity confidence interval, and used the upper 95% confidence level as a threshold for classifying MID deficits. The upper 95% confidence level was 0.32 for the monocular cue condition and 0.22 for the binocular and combined cue conditions. The threshold was higher in the monocular cue condition because each eye saw half the number of trials presented in the binocular and combined cue conditions. For simplicity, we used the slightly less conservative 0.32 deficit threshold for all cue conditions, which led to the classification of one additional binocular cue deficit.

We used linear mixed effects (LME) models to test for relationships between cue conditions and generalized linear mixed effects (GLME) models to compare sensitivities between groups (e.g., ipsilateral vs. contralateral eye, or different cue conditions). Visual field location and sensitivity were treated as fixed effects, and observer was treated as a random effect. For the GLME models, groups were treated as fixed effects. We applied Bonferroni correction in all analyses that simultaneously tested multiple hypotheses.

## Results

### Binocular MID cue sensitivity varies across eccentricity-matched visual field locations

**Figure 2:**
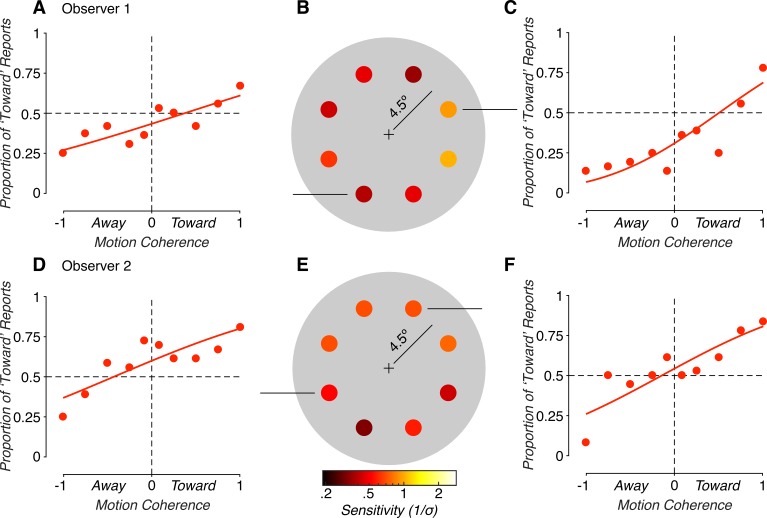
Motion-in-depth discrimination based on binocular cues. (A) Proportion of “toward” reports as a function of motion coherence (proportion of signal dots), at a single visual field location. Positive (negative) coherences indicate that the dots moved toward (away). Solid curves are cumulative Gaussian fits. (B) Sensitivity at all eight tested visual field locations. Darker colors indicate smaller sensitivities, lighter colors indicate greater sensitivities. The fixation point is marked by a plus sign. (C) Performance at a second visual field location. (D–F) Same format as A–C for a second observer. Both observers showed considerable variability in sensitivity to binocular MID cues across the visual field.

We first evaluated how sensitivity to binocular MID cues varied across eccentricity-matched locations of the visual field. The proportion of “toward” responses as a function of direction and motion coherence are shown for two visual field locations of a representative observer in Figure 2A and C. In some locations, sensitivity ($\newcommand{\bialpha}{\boldsymbol{\alpha}}\newcommand{\bibeta}{\boldsymbol{\beta}}\newcommand{\bigamma}{\boldsymbol{\gamma}}\newcommand{\bidelta}{\boldsymbol{\delta}}\newcommand{\bivarepsilon}{\boldsymbol{\varepsilon}}\newcommand{\bizeta}{\boldsymbol{\zeta}}\newcommand{\bieta}{\boldsymbol{\eta}}\newcommand{\bitheta}{\boldsymbol{\theta}}\newcommand{\biiota}{\boldsymbol{\iota}}\newcommand{\bikappa}{\boldsymbol{\kappa}}\newcommand{\bilambda}{\boldsymbol{\lambda}}\newcommand{\bimu}{\boldsymbol{\mu}}\newcommand{\binu}{\boldsymbol{\nu}}\newcommand{\bixi}{\boldsymbol{\xi}}\newcommand{\biomicron}{\boldsymbol{\micron}}\newcommand{\bipi}{\boldsymbol{\pi}}\newcommand{\birho}{\boldsymbol{\rho}}\newcommand{\bisigma}{\boldsymbol{\sigma}}\newcommand{\bitau}{\boldsymbol{\tau}}\newcommand{\biupsilon}{\boldsymbol{\upsilon}}\newcommand{\biphi}{\boldsymbol{\phi}}\newcommand{\bichi}{\boldsymbol{\chi}}\newcommand{\bipsi}{\boldsymbol{\psi}}\newcommand{\biomega}{\boldsymbol{\omega}}1/\sigma$) was well above the 0.32 threshold defining chance performance. For example, at the location shown in Figure 2C, the observer's sensitivity was 0.96. In other locations, sensitivity approached chance levels (e.g., 0.44 in Figure 2A). As summarized in Figure 2B, sensitivity varied considerably across the tested visual field locations for this observer: Sensitivity ranged from 0.39 to 1.04, and thus varied more than two-fold (maximum/minimum sensitivity). Variability in binocular cue sensitivity across the visual field was not unique to this observer. Performance for a second observer is shown in Figure 2 (bottom row). For this observer, sensitivity ranged from 0.36 to 0.83, and thus also varied more than two-fold. Across the seven observers, there was an average 2.57 ± 0.41-fold *SEM* difference in sensitivity across the tested visual field locations.

Previous studies found that some observers are unable to discriminate the direction of MID based on binocular cues in certain idiosyncratic regions of the visual field despite otherwise normal vision in those regions (Barendregt et al., 2014, 2016; Hong & Regan, 1989). In the current data set, we found that one observer showed chance performance with binocular MID cues in two neighboring visual field locations. Since those two locations were at the same eccentricity as the nondeficit locations, and the retinal speeds of the stimuli were equivalent, the difference in performance (ranging from 0.2 to 0.98, nearly five-fold) cannot be explained by viewing geometry, and therefore suggests a neural basis. The current results are thus consistent with previous findings of highly variable binocular MID discrimination across the visual field, including deficits in binocular MID cue processing for some observers.

### Monocular MID cue sensitivity depends on the visual field location relative to the stimulated eye

**Figure 3:**
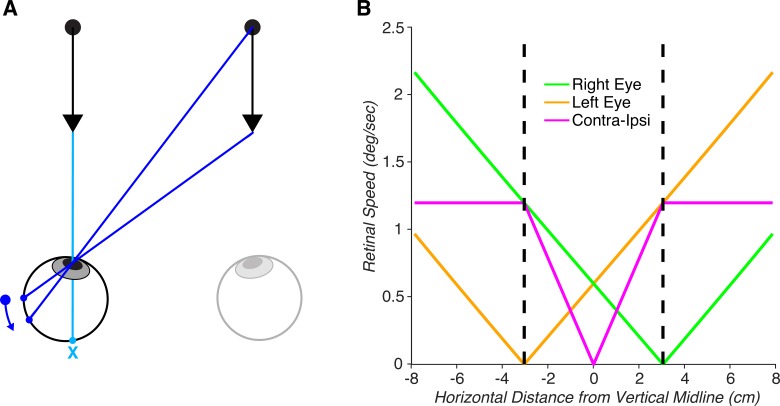
Motion-in-depth stimuli produce different retinal motion signals in the two eyes. (A) The retinal motion signal produced by an MID stimulus depends on the lateral distance of the stimulus from the eye. An MID stimulus directly in front of the left eye produces a 0 speed signal in that eye (light blue X; ignoring looming). An MID stimulus directly in front of the right eye produces a nonzero retinal motion signal in the left eye (dark blue arrow). (B) Retinal speed as a function of position of an MID stimulus relative to the vertical midline for the right (green) and left (orange) eyes. These values are based on a disparity range of ±0.15° (90 cm viewing distance), a 250 ms duration, and an IOD of 6.1 cm. Also plotted is the contralateral–ipsilateral difference in retinal motion speeds (magenta). As can be seen from this curve, retinal motion speed is greater in the contralateral eye than the ipsilateral eye.

An MID stimulus produces different retinal signals in the two eyes. In the binocular MID literature, this is appreciated as producing interocular velocity differences. What is less appreciated, is that MID stimuli also produce two distinct monocular (optic flow) patterns. This becomes clear when considering stimuli presented away from the vertical midline (Figure 3A). A single point moving directly toward the left eye will produce a zero-velocity signal on the left retina (ignoring looming cues), but a nonzero velocity signal on the right retina. The relationship between stimulus location and retinal speed is illustrated in Figure 3B. This calculation assumed a point moving perpendicular to the plane of fixation as in Figure 3A, a disparity range of ±0.15° (with a 90 cm viewing distance), a 250 ms duration, and an interocular distance (IOD) of 6.1 cm. The pattern was qualitatively similar over a wide range of viewing distances and disparity ranges. For each eye, retinal speed as a function of horizontal position is a V-shaped curve, with a minimum (0 speed) when the stimulus is located directly in front of the eye. Also plotted is the contralateral–ipsilateral difference in retinal speeds (magenta curve). The retinal speed difference increases from the vertical midline (where the difference is zero) to half the IOD (where the stimulus is located directly in front of one of the eyes). Beyond that point, the difference in retinal motion speeds is constant. This illustrates that stimuli which move perpendicular to the plane of fixation over a fixed range of horizontal disparities produce larger retinal motion signals in the contralateral eye compared to the ipsilateral eye.

**Figure 4:**
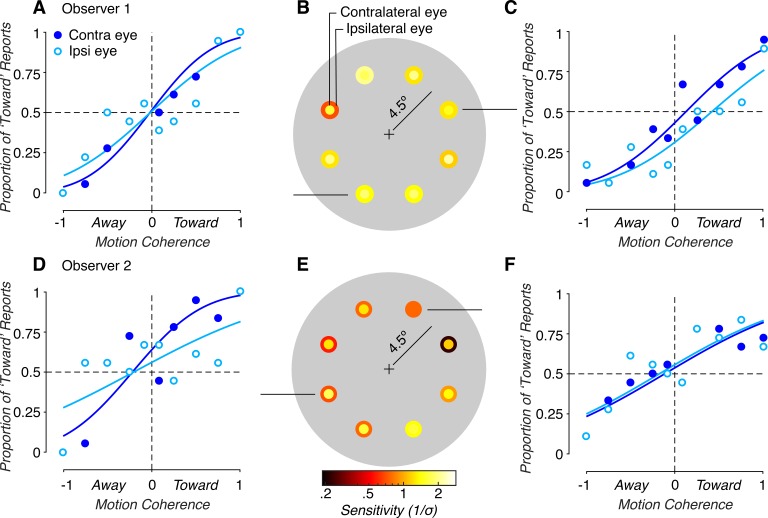
Motion-in-depth discrimination based on monocular cues. Data are from the same observers and visual field locations shown in Figure 2. (A), (C) and (D), (F) Monocular cue performance at individual visual field locations for contralateral eye (dark blue) and ipsilateral eye (light blue) stimulus presentations. (B) and (E) Contralateral and ipsilateral sensitivities at all tested visual field locations. Darker colors indicate smaller sensitivities, and lighter colors indicate greater sensitivities. The color of the inner (outer) circle denotes sensitivity in the contralateral (ipsilateral) eye. For the two representative observers, contralateral sensitivity was greater than the ipsilateral sensitivity at all but one visual field location (112.5°, ∼11 o'clock on a clockface, for observer 1). The four visual field locations closest to the vertical midline (e.g., panels A and F) are within the IOD (between the dashed lines in Figure 3B), where the difference in retinal speeds is smaller. The four locations furthest from the vertical midline (e.g., panels C and D) are outside the IOD, where the difference in retinal speeds is maximal.

**Figure 5:**
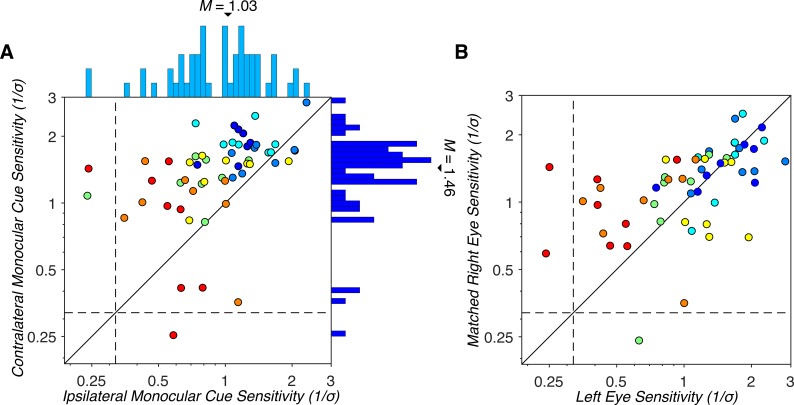
Comparison of monocular cue sensitivities. (A) Comparison of contralateral and ipsilateral monocular cue sensitivities. Each point represents one observer's sensitivity at a single visual field location (N = 56 points). Each color represents one observer (N = 7). Marginal histograms show the distributions of sensitivities. The majority of points lay above the identity line (solid black diagonal), indicating greater sensitivity to stimuli presented to the contralateral eye than the ipsilateral eye. Horizontal and vertical black dashed lines indicate the upper boundaries of chance performance. MID deficits are indicated by points that fall below or left of the dashed black lines. (B) Comparison of right and left eye monocular cue sensitivities for stimuli with matched optic flow patterns. For most observers, the data are distributed along the identity line, suggesting that viewing geometry accounts for a major component of the observed variability in monocular cue sensitivity across the visual field.

Following these geometric considerations, we assessed monocular cue sensitivity across the visual field for the right and left eyes separately. We then grouped the results based on whether the stimulus was presented to the contralateral or ipsilateral eye. Performance with contralateral and ipsilateral monocular cues is plotted in Figure 4 for the same observers and visual field locations shown in Figure 2. If observers are sensitive to the differences in optic flow patterns in the two eyes, they should perform best with stimuli presented in the eye contralateral to the stimulus location. This was indeed what we found. For both observers, sensitivities were greater in the contralateral than in the ipsilateral eye in almost all tested visual field locations (Figure 4B and E). Sensitivity to contralateral versus ipsilateral monocular MID cues (contra/ipsi) showed an average 1.56 ± 0.14-fold *SEM* difference for observer 1 and a 1.95 ± 0.39-fold *SEM* difference for observer 2. Other observers showed similar results. Sensitivity was greatest when the stimuli were presented to the contralateral eye in the vast majority of cases (Figure 5A). Across all observers and visual field locations, contralateral monocular cue sensitivity (*M* = 1.46, *SD* = 0.51) was significantly greater than ipsilateral monocular cue sensitivity (*M* = 1.03, *SD* = 0.47); GLME: *b* = 0.43, *F*(1, 109) = 41.47, *p* = 3.31 × 10^−9^. This result indicates that sensitivity to monocular MID cues depends on the visual field location of the stimulus relative to the stimulated eye.

We further found that sensitivity to both contralaterally and ipsilaterally presented monocular MID stimuli varied considerably across the visual field. For stimuli presented to the contralateral eye, sensitivity across the tested visual field locations varied 2.8 ± 0.67-fold *SEM* on average (*N* = 7 observers). Likewise, for stimuli presented to the ipsilateral eye, sensitivity varied 3.14 ± 0.41-fold *SEM* on average (*N* = 7 observers). One observer was found to exhibit chance performance for stimuli presented to the left eye at two visual field locations (one ipsilateral and one contralateral). Another observer exhibited chance performance at a single visual field location for stimuli presented to the right eye at an ipsilateral visual field location. These findings indicate that large variability in MID sensitivity across eccentricity-matched visual field locations and MID processing deficits are not unique to binocular cues, but also occur with monocular cues.

To assess the contribution of viewing geometry to monocular cue sensitivity, we first compared right and left eye sensitivities to stimuli with matched optic flow patterns. Specifically, we compared right and left eye sensitivities at stimulus locations mirrored about the vertical midline (e.g., the lower left aperture viewed through the right eye, and the lower right aperture viewed through the left eye). When the optic flow patterns were matched across the two eyes, the sensitivities were similar (Figure 5B), and left eye sensitivity significantly predicted the matched right eye sensitivity, *b* = 0.46, *F*(1, 53) = 29.07, *p* = 1.65 × 10^−6^. This result is consistent with the geometric analysis in Figure 3B which shows that absolute differences in retinal motion speeds are symmetric about the vertical midline.

We next compared differences in sensitivities at visual field locations “near” versus “far” from the vertical midline. At near locations (within the IOD), the difference in contralateral and ipsilateral retinal speeds was 1.07°/s at the aperture centers (see Figure 3B). At far locations (outside the IOD), the difference in retinal speeds was maximal (1.2°/s). We found that the difference in contralateral and ipsilateral sensitivities depended on the difference in retinal speeds. At near locations, the average difference between contralateral and ipsilateral sensitivities was 0.23 ± 0.08 *SEM*; *N* = 28 (4 locations × 7 observers). At far locations, the average difference was 0.62 ± 0.08 *SEM*; *N* = 28. A GLME confirmed that there was a significant interaction between the stimulated eye (contralateral vs. ipsilateral) and distance from the vertical midline (near vs. far); GLME: *b* = 0.39, *F*(1, 108) = 9.32, *p* = 2.85 × 10^−3^. These results suggest that viewing geometry was a major component underlying variability in monocular MID cue sensitivity across the visual field. However, a neural contribution was also evident since monocular MID deficits were found at the same eccentricity as nondeficit locations, and some sensitivity differences persisted for stimuli with matched optic flow patterns (Figure 5B).

### Comparison of binocular and monocular MID cue sensitivities

**Figure 6:**
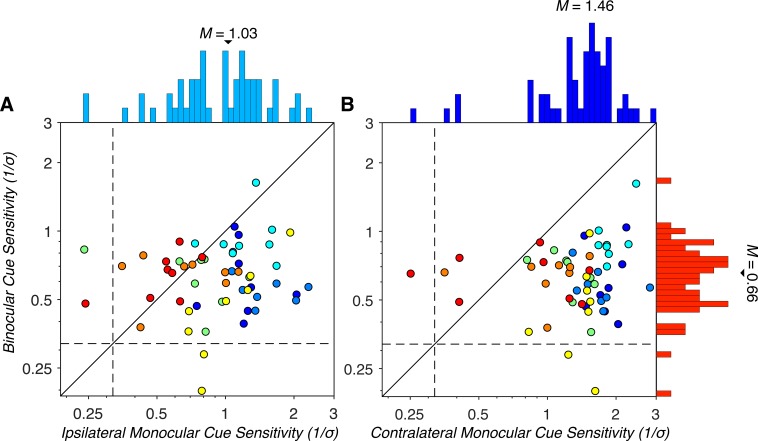
Comparison of binocular and monocular MID cue sensitivities. Plotted as in Figure 5. (A) Binocular versus ipsilateral monocular cue sensitivities. (B) Binocular versus contralateral monocular cue sensitivities. In both panels, the majority of points fall below the identity line, indicating that the observers were generally more sensitive to monocular cues than to binocular cues. Three observers showed MID deficits. One showed binocular MID deficits, and two showed monocular MID deficits. None of the observers showed deficits for both cue types.

Having characterized binocular and monocular MID cue sensitivities individually, we next compared sensitivities to the two cue types. Binocular and monocular cue sensitivities are plotted against each other for all visual field locations and observers in Figure 6. Since contralateral and ipsilateral monocular cue sensitivities differed, we separately compared each to the binocular cue sensitivities. Both contralateral (*M* = 1.46, *SD* = 0.51) and ipsilateral (*M* = 1.03, *SD* = 0.47) monocular cue sensitivities were significantly greater than binocular cue sensitivities (*M* = 0.66, *SD* = 0.23); GLME: *b* = 0.80, *F*(1, 109) = 162.20, *p* = 2.61 × 10^−23^ (contralateral), and *b* = 0.37, *F*(1, 109) = 36.50, *p* = 2.16 × 10^−8^ (ipsilateral). Thus, observers were generally better at discriminating the direction of MID based on monocular cues than binocular cues. Furthermore, binocular cue sensitivity could not be predicted from either contralateral; LME: *b* = 0.07, *F*(1, 53) = 1.05, *p* = 0.31, or ipsilateral, *b* = 0.07, *F*(1, 53) = 0.94, *p* = 0.34, monocular cue sensitivity. This result may reflect that the processing of binocular and monocular cues depends, at least partially, on separate neural mechanisms. We further found that at all tested visual field locations, all observers performed significantly above chance in at least one of the cue-isolated conditions.

### Comparison of combined cue and cue-isolated MID sensitivities

**Figure 7:**
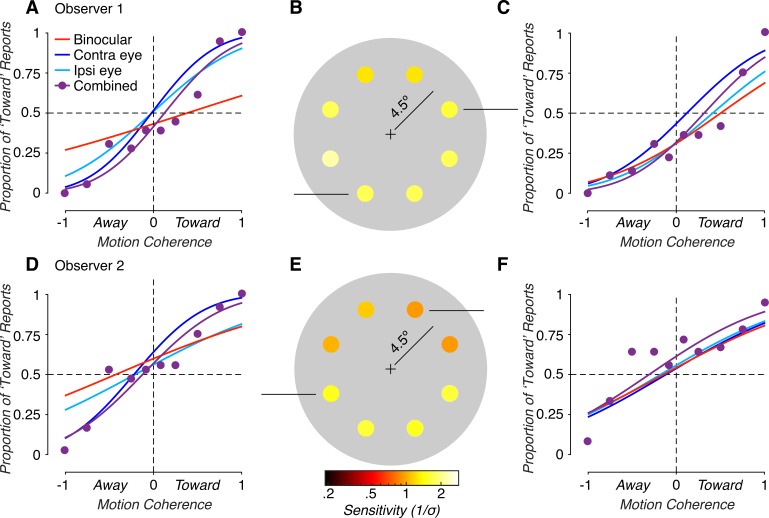
Motion-in-depth discrimination based on combined (binocular and monocular) cues. Data are from the same observers and visual field locations shown in Figures 2 and 4. (A), (C) and (D), (F) Combined cue performance at individual visual field locations (purple). Psychometric fits for the binocular (red), contralateral monocular (dark blue), and ipsilateral monocular (light blue) cue conditions are shown for comparison. (B) and (E) Combined cue sensitivities at all tested visual field locations. Darker colors indicate smaller sensitivities, and lighter colors indicate greater sensitivities. Considerable variability in MID discrimination across the visual field remained evident even when both binocular and monocular cues were present.

We next assessed sensitivity to MID stimuli that contained both binocular and monocular cues. Figure 7 shows combined cue performance for the same observers and visual field locations shown in Figures 2 and 4. For comparison, psychometric fits from the cue-isolated conditions are also shown. Sensitivity to combined cue stimuli varied considerably across the tested visual field locations for both representative observers. For observer 1, sensitivity ranged from 1.18 to 2.16, and thus varied 1.83-fold. For observer 2, sensitivity ranged from 0.94 to 1.52, and thus varied 1.62-fold. Across the seven observers, there was an average 1.91 ± 0.20-fold *SEM* difference in sensitivity across the tested visual field locations. Thus, while variability in MID discrimination across the visual field was smaller in the combined cue condition than in any of the cue-isolated conditions, large variability remained evident.

**Figure 8:**
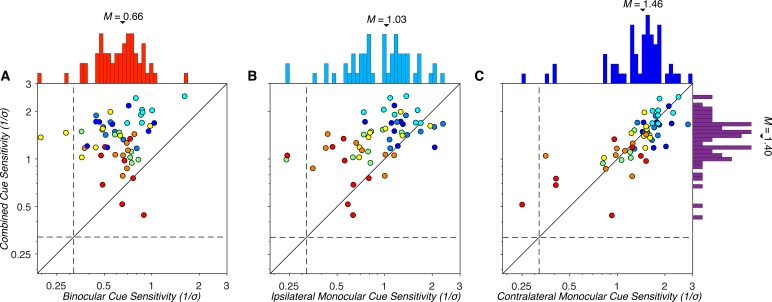
Comparison of combined cue and cue-isolated MID sensitivities. Plotted as in Figures 5 and 6. (A) Combined versus binocular cue sensitivities. All but three points lay above the identity line, indicating greater sensitivity to combined cue stimuli than binocular cue stimuli. (B) Combined versus ipsilateral monocular cue sensitivities. The majority of points lay above the identity line, indicating greater sensitivity to combined cue stimuli than ipsilateral monocular cue stimuli. (C) Combined versus contralateral monocular cue sensitivities. The data points are largely distributed along the identity line, indicating that contralateral monocular cue sensitivity was a strong predictor of combined cue sensitivity.

Lastly, we compared combined cue sensitivity to binocular and monocular cue sensitivities across observers and visual field locations. Combined cue sensitivity (*M* = 1.40, *SD* = 0.44) was significantly greater than both binocular (*M* = 0.66, *SD* = 0.23), GLME: *b* = −0.74, *F*(1, 109) = 198.89, *p* = 2.50 × 10^−26^ (Figure 8A), and ipsilateral monocular (*M* = 1.03, *SD* = 0.47), GLME: *b* = −0.37, *F*(1, 109) = 36.45, *p* = 2.20 × 10^−8^ (Figure 8B), cue sensitivities. However, we did not find a significant difference between combined cue sensitivity and contralateral monocular cue sensitivity (*M* = 1.46, *SD* = 0.51), GLME: *b* = 0.06, *F*(1, 109) = 1.01, *p* = 0.32 (Figure 8C). We further tested if combined cue sensitivity could be predicted from any of the cue-isolated sensitivities. Combined cue sensitivity was not significantly predicted by binocular, LME: *b* = 0.16, *F*(1, 53) = 0.72, *p* = 0.40, or ipsilateral monocular, LME: *b* = 0.09, *F*(1, 53) = 0.76, *p* = 0.39, cue sensitivities. However, combined cue sensitivity was significantly predicted by contralateral monocular cue sensitivity, LME: *b* = 0.47, *F*(1, 53) = 29.38, *p* = 1.49 × 10^−6^. These results suggest that contralateral monocular cues provide a substantial source of information for observers to judge the direction of MID.

## Discussion

In this study, we assessed the contributions of binocular and monocular cues to MID perception. Sensitivity to monocular cues generally exceeded sensitivity to binocular cues. For both cue types, we found substantial variability in sensitivity across the visual field. However, the sources of variability differed for the two cues. For binocular cues, we found variability in sensitivity to stimuli matched for eccentricity and retinal speed, suggesting a neural basis. While a neural contribution to variability in monocular MID cue sensitivity across the visual field likely also existed, another major source of variability was geometric. MID off of the vertical midline and moving perpendicular to the plane of fixation produces larger retinal motion signals in the eye contralateral to the visual field location of the stimulus. This resulted in greater MID sensitivity for stimuli presented to the contralateral eye than the ipsilateral eye. We further found that contralateral monocular cues were a strong predictor of combined cue sensitivity. These results identify distinct factors constraining the contributions of binocular and monocular cues to 3D motion perception, and have fundamental implications for understanding how signals from the two eyes are combined to create 3D motion representations.

### Contributions of binocular cues to MID perception

Sensitivity to binocular MID cues varied greatly across eccentricity-matched visual field locations. This variability could not be attributed to viewing geometry since the retinal speeds were the same at all locations. As such, the observed variability likely had a neural basis. In particular, this variability may reflect differences in the efficacy of binocular integration of retinal motion signals at different locations in the visual field.

Previous work found that binocular MID cue sensitivity can be particularly poor, such that as many as 60% of observers are unable to discriminate the direction of MID in discrete locations of the visual field, despite being able to make accurate judgments of position in depth and having no difficulties seeing stimuli at those locations (Barendregt et al., 2014, 2016; Hong & Regan, 1989; Richards & Regan, 1973). However, in the present study, only one of the seven observers had severely impoverished performance in the binocular cues condition. What might account for this difference? The current study differed from prior work investigating binocular MID deficits in several ways (Barendregt et al., 2014, 2016). First, we used higher contrast stimuli, which may have reduced the difficulty of the task since 3D motion processing is more sensitive to contrast (Fulvio, Rosen, & Rokers, 2015) than might be expected based on two-dimensional motion perception studies (Weiss, Simoncelli, & Adelson, 2002). Second, the aperture size and number of dots used in the present study were larger, reducing task difficulty. Third, we reduced apparent MID signals in the direction opposite of the intended stimulus direction by presenting the dots within a volume rather than on a plane. Fourth, we used a different “diagnostic” criterion for identifying MID deficits. We varied motion coherence, and bootstrapped a sensitivity value that corresponded to chance performance, as opposed to performing a binomial test. These changes aided the goal of assessing the contributions of binocular and monocular cues to MID perception, but may have reduced the ability to detect MID deficits. Future work with a larger sample could establish an accurate prevalence rate of binocular MID deficits, and determine if differences across studies are due to stimuli, diagnostic criteria, or observer variability.

### Contributions of monocular cues to MID perception

We found that monocular cue sensitivity was also highly variable across the visual field. The geometry of 3D viewing dictates that MID stimuli moving perpendicular to the plane of fixation over a fixed horizontal disparity range will produce larger retinal motion signals in the contralateral eye than in the ipsilateral eye. As such, we considered the possibility that monocular sensitivity to MID stimuli might differ between the two eyes as a function of stimulus location. By using stimuli that preserved the natural differences in optic flow patterns in each eye, we found greater sensitivity to MID stimuli presented to the contralateral eye than the ipsilateral eye. Furthermore, the difference between right and left eye sensitivities was larger when the difference between retinal speeds in the two eyes was greater. When right and left eye sensitivities were compared at locations with matched optic flow patterns, sensitivity was generally well matched. These findings indicate a large geometric component to variability in monocular cue sensitivity across the visual field.

Our approach differed from previous work on monocular MID cue processing which presented the same stimulus pattern to both eyes simultaneously (Cottereau et al., 2017; de Jong et al., 1994; Duffy & Wurtz, 1991a; Graziano et al., 1994; Mineault et al., 2012; Morrone et al., 2000; Uesaki & Ashida, 2015; Xu et al., 2014). Such stimuli assume a “cyclopean eye,” ignoring the interocular distance that results in different optic flow patterns on each retina (Cormack et al., 2017). Rendering based on a cyclopean eye does not provide a faithful representation of monocular inputs under naturalistic conditions, and can potentially impact neural responses. Likewise, binocular viewing of stimuli that isolate monocular cues can impact neural responses. For example, 3D surface orientation selective neurons in parietal cortex are sensitive to the difference between monocular and binocular viewing of monocular cue stimuli (Rosenberg & Angelaki, 2014). Binocular viewing of such stimuli introduces a cue conflict when the monocular cues signal a stimulus extending in depth since the binocular disparities signal a stimulus confined to the plane of fixation. Reflecting that cue conflict, neuronal responses to combined cue stimuli were more similar to responses to monocular cue stimuli that were viewed monocularly than binocularly. Together with the current study, these results suggest that differences between (a) right and left eye signals, and (b) binocular versus monocular viewing cannot be ignored when assessing monocular cue sensitivity.

### Comparing binocular, monocular, and combined cue sensitivities

Contralateral and ipsilateral monocular cue sensitivities were both significantly greater than binocular cue sensitivity. Combined cue sensitivity was significantly greater than both binocular and ipsilateral monocular cue sensitivities. However, contralateral monocular cue and combined cue sensitivities were highly similar. One interpretation of these results is that MID perception relies largely on contralateral monocular signals, but there might be another explanation. Standard cue integration models predict that combined cue sensitivity will be similar to the best isolated cue when the individual cue sensitivities differ substantially (Landy, Banks, & Knill, 2014), as was the case here. The evaluation of cue integration for MID perception will therefore require cue-isolated stimuli that produce more balanced sensitivities.

Previous studies found that some observers are unable to discriminate the direction of MID based on binocular cues. These MID deficits exist in idiosyncratic and circumscribed regions of the visual field in which vision appears otherwise normal (Barendregt et al., 2014, 2016; Hong & Regan, 1989). Here we found that some observers show similar MID deficits for monocular cues. In the current data set, we found no cases in which binocular and monocular MID cue deficits overlapped. This suggests that different MID cues can help compensate for deficits in the processing of other cues.

### Implications for the neural basis of MID perception

Binocular and monocular MID cue sensitivities were statistically independent, and deficits in the processing of the two cue types did not overlap spatially. These findings are consistent with the distinct computations required to extract MID information from these cues (Lages & Heron, 2010; Longuet-Higgins & Prazdny, 1980; Koenderink, 1986), and that the two cues appear to be processed, at least partially, within different cortical areas. Since MID deficits at a given visual field location were cue-specific, it is possible that binocular and monocular MID deficits originate within different neuronal populations that separately process the two cue types.

Selectivity for binocular MID cues has been observed in macaque MT (Czuba, Huk, Cormack, & Kohn, 2014; Sanada & DeAngelis, 2014) and the human analogue hMT+ (Rokers, Cormack, & Huk, 2009). However, macaque MT neurons do not show clear selectivity for monocular optic flow patterns (Lagae, Maes, Raiguel, Xiao, & Orban, 1994). In contrast, robust responses to monocular MID cues are found in macaque MST (Duffy & Wurtz, 1991b; Raiguel et al., 1997) and VIP (Bremmer, Duhamel, Ben Hamed, & Graf, 2002; Sunkara, DeAngelis, & Angelaki, 2015). Thus, MT and MST/VIP may contribute differently to MID discrimination when stimuli are defined by binocular or monocular cues. For small stimuli, like those used in the current study, the ventral/lateral subdivision of MST may be particularly important for MID discrimination based on monocular cues. Neurons in this subdivision of MST have relatively small receptive fields compared to dorsal MST or VIP, and may support object-motion processing as opposed to self-motion processing (Eifuku & Wurtz, 1998; Tanaka, Sugita, Moriya, & Saito, 1993). Lastly, we found that the visual system is sensitive to differences in the right and left eye optic flow patterns produced by MID stimuli. The current results thus raise the question of *how* the two optic flow patterns are integrated at the neuronal level to support MID perception.

### 3D perception using stereoscopic displays

A concern in vision research is the extent to which virtually rendered 3D stimuli elicit realistic percepts. Here we asked observers to report the perceived direction of MID (toward vs. away). However, for monocular cues, it is possible to adopt a heuristic and report radial motion away from the fixation point as “toward” and radial motion toward the fixation point as “away.” Regardless of whether such stimuli can elicit non-MID percepts, it is unlikely that the observers relied on such a heuristic. First, they were not given feedback, so they could not have learned a strategy based on reward contingencies. If they spontaneously adopted a strategy, we would expect some to adopt the opposite heuristic: indicating radial motion away from the fixation point as “away” and radial motion toward as “toward.” This would have resulted in inverted psychometric functions, which were not observed. Second, the heuristic would not work for the binocular cue stimuli since they lacked radial motion. Observers would therefore have needed to maintain different heuristics for the two cue-isolated conditions. Third, how would those two heuristics interact in the combined cue condition? Assume that the observers followed the instruction to report the perceived direction of MID (above chance performance with binocular cue stimuli indicates this was true). If the monocular cues did not elicit MID percepts, the combined cue sensitivities would be expected to match the binocular cue sensitivities. Instead, they matched the contralateral monocular cue sensitivities. This suggests that either (a) the monocular cue stimuli elicited MID percepts or (b) the observers selectively ignored the task instructions for monocular and combined cue stimuli. Together, these observations suggest it was more likely that observers based their responses on MID percepts rather than response heuristics.

Another concern in 3D vision research is that sensitivity in experimental settings is relatively poor compared to our experiences in the world. This may reflect that sensory signals which contribute to perception in natural conditions are not necessarily present in an experiment. For example, in addition to binocular and monocular cues studied here, 3D perception depends on cues such as lighting (shading), self-motion (motion parallax), and ocular cues (blur and accommodation; Buckthought, Yoonessi, & Baker, 2017; de la Malla, Buiteman, Otters, Smeets, & Brenner, 2016; Muryy, Welchman, Blake, & Fleming, 2013; Zannoli, Love, Narain, & Banks, 2016). Recent advances in 3D rendering and display technologies have increased the ability to study perception under more naturalistic conditions, and promise to yield further insights into how individual sensory cues contribute to 3D perception.

Prior experience with 3D displays also affects perception of 3D rendered stimuli. For example, some observers seem to initially discount binocular cues, perhaps because the majority of encountered displays only contain two-dimensional cues. Task feedback seems especially helpful in improving the use of cues available in 3D displays (Fulvio & Rokers, 2017). Further work is needed to better understand the mechanisms that facilitate the use of certain sensory cues that signal 3D information through experience and feedback.

## Supplementary Material

Supplement 1

Supplement 2

Supplement 3

Supplement 4

## Supplementary material

**Supplementary Movie S1.** Example combined cue stimulus.

**Supplementary Movie S2.** Example contralateral monocular cue stimulus.

**Supplementary Movie S3.** Example ipsilateral monocular cue stimulus.

**Supplementary Movie S4.** Example binocular cue stimulus.
